# Endometrial regeneration with endometrial epithelium: homologous orchestration with endometrial stroma as a feeder

**DOI:** 10.1186/s13287-021-02188-x

**Published:** 2021-02-12

**Authors:** Ryo Yokomizo, Yukiko Fujiki, Harue Kishigami, Hiroshi Kishi, Tohru Kiyono, Sanae Nakayama, Haruhiko Sago, Aikou Okamoto, Akihiro Umezawa

**Affiliations:** 1 Center for Regenerative Medicine, National Center for Child Health and Development Research Institute, 2-10-1 Okura, Setagaya, Tokyo, 157-8535 Japan; 2 Center for Maternal-Fetal, Neonatal and Reproductive Medicine, National Center for Child Health and Development, 2-10-1 Okura, Setagaya, Tokyo, 157-8535 Japan; 3 Department of Obstetrics and Gynecology, The Jikei University School of Medicine, 3-25-8 Nishi-Shinbashi, Minato, Tokyo, 105-8461 Japan; 4 Project for Prevention of HPV-related Cancer, Exploratory Oncology Research and Clinical Trial Center, National Cancer Center, Chiba, 277-8577 Japan

**Keywords:** Endometrial regeneration, Endometrial epithelium, Endometrial stroma, Human embryonic stem cell, Three-dimensional culture

## Abstract

**Background:**

Thin endometrium adversely affects reproductive success rates with fertility treatment. Autologous transplantation of exogenously prepared endometrium can be a promising therapeutic option for thin endometrium; however, endometrial epithelial cells have limited expansion potential, which needs to be overcome in order to make regenerative medicine a therapeutic strategy for refractory thin endometrium. Here, we aimed to perform long-term culture of endometrial epithelial cells in vitro.

**Methods:**

We prepared primary human endometrial epithelial cells and endometrial stromal cells and investigated whether endometrial stromal cells and human embryonic stem cell-derived feeder cells could support proliferation of endometrial epithelial cells. We also investigated whether three-dimensional culture can be achieved using thawed endometrial epithelial cells and endometrial stromal cells.

**Results:**

Co-cultivation with the feeder cells dramatically increased the proliferation rate of the endometrial epithelial cells. We serially passaged the endometrial epithelial cells on mouse embryonic fibroblasts up to passage 6 for 4 months. Among the human-derived feeder cells, endometrial stromal cells exhibited the best feeder activity for proliferation of the endometrial epithelial cells. We continued to propagate the endometrial epithelial cells on endometrial stromal cells up to passage 5 for 81 days. Furthermore, endometrial epithelium and stroma, after the freeze-thaw procedure and sequential culture, were able to establish an endometrial three-dimensional model.

**Conclusions:**

We herein established a model of in vitro cultured endometrium as a potential therapeutic option for refractory thin endometrium. The three-dimensional culture model with endometrial epithelial and stromal cell orchestration via cytokines, membrane-bound molecules, extracellular matrices, and gap junction will provide a new framework for exploring the mechanisms underlying the phenomenon of implantation. Additionally, modified embryo culture, so-called “in vitro implantation”, will be possible therapeutic approaches in fertility treatment.

**Supplementary Information:**

The online version contains supplementary material available at 10.1186/s13287-021-02188-x.

## Background

Infertility is defined as a failure to conceive after 12 months of regular and unprotected sexual intercourse and is estimated to affect 8–12% of couples of reproductive age [1]. Various etiologies are associated with infertility, and endometrium is recognized as one of the significant factors [2]. Endometrium is structurally divided into two indistinct layers, epithelial cells and stromal cells, and undergoes remarkable histological and structural changes throughout the menstrual cycle in preparation for embryonic implantation and subsequent shedding and regeneration in non-conception cycles. Endometrial thickness and a sonographic pattern of the endometrium are widely accepted as prognostic indicators for endometrial receptivity; lower endometrial thickness is associated with lower pregnancy rate and live birth rate [3]. Regardless of its significance, gynecologists often perform dilatation and curettage for spontaneous miscarriage and abortion [4], and interventional radiologists perform embolization for feeding arteries to control vaginal bleeding or reducing size of uterine myoma in clinical practice [5]; nevertheless, these invasive interventions cause endometrial damage and result in thin endometrium [6, 7].

Thin endometrium adversely affects reproductive success rates with fertility treatment [8]. Several treatment modalities are presented to patients with thin endometrium to improve endometrial thickness [9]. These approaches comprise estradiol replacement, supplementation of elemental minerals and vitamins, and uncountable experimental approaches. Regenerative medicine including application of platelet-rich plasma (PRP) and cell therapy utilizing menstruation-derived stem cells is expected to be a promising therapeutic strategy [10, 11]. However, these strategies are not broadly available and there is no reliable clinical evidence supporting their use. Autologous transplantation of exogenously prepared endometrium is currently a challenge for clinical practice [12]. In addition, endometrial organoid with endometrial cells is established [13], but endometrial epithelial cells themselves have not been cultured in vitro. Considering these limitations, further study is needed to determine how to maintain endometrial epithelial cells efficiently under xeno-free conditions.

We herein established a model of in vitro-cultured endometrium as a potential therapeutic option for refractory thin endometrium. The three-dimensional culture model with endometrial epithelial and stromal cell orchestration will provide a new framework for exploring the mechanisms underlying the phenomenon of implantation. Additionally, modified embryo culture, so-called “in vitro implantation”, will be possible therapeutic approaches in fertility treatment.

## Methods

### Human tissue collection

Endometrial specimens without any abnormalities or malignancies were obtained from women of reproductive age undergoing hysterectomy for benign gynecological diseases after written informed consent. Characteristics of donor individuals are provided in Table 1.

**Table 1:** Patients’ information

Parameters	Mean ± SD
Age	47.0 ± 2.8
BMI	23.4 ± 4.1
BMI, body mass index; SD, standard deviation	
Parameters	N
Gynecological disorder (including duplication)	
Uterine myoma	9
Endometriosis	3
Ovarian tumor	2
Adenomyosis	1
Operation	
Abdominal hysterectomy	5
Laparoscopic hysterectomy	4
Menstrual regularity	
Regular	3
Irregular	4
Unknown	2
Menstrual phase	
Proliferative	5
Secretory	2
Unknown	2

Endometrial epithelial cells and stromal cells were isolated from endometrial tissues as reported previously [14]. Briefly, after enzymatic digestion of minced tissue with 100 μg/ml collagenase type 1 in a shaking incubator for 2 h at 37 °C, cells were separated by filtration through 100 μm and 40 μm nylon mesh. The dispersed fragments were collected by centrifugation, resuspended in DMEM-high glucose (Gibco, catalog number 11965-118) containing 1% penicillin-streptomycin (Gibco, catalog number 15140-122) and 10% fetal bovine serum (Gibco, catalog number 16000-044) and seeded on biocoat culture dishes (Corning, catalog number 354450) as endometrial stromal cells. The residual tissue fragments and cell clumps were collected into a new 50-ml tube using Accumax (Innovative Cell Technologies, catalog number AM105) and 0.25% Trypsin/EDTA (Gibco, catalog number 25200-056) and then incubated for 10 min at room temperature with continuous pipetting. Cells separated by filtration through a 40-μm nylon mesh, which were regarded as endometrial epithelial cells, were resuspended in ESTEM-HE medium (GlycoTechnica, Japan) and seeded on culture dishes. Portions of endometrial epithelial cells were frozen with Stem Cellbanker (Nippon Zenyaku Kogyo, Japan) in − 80 °C. Endometrial stromal cells and epithelial cells were incubated at 37 °C, 95% air and 5% CO_2_. These cells were passaged serially when they reached confluent by using TrypLE Express (Gibco, catalog number 12605-010) and frozen with STEM CELLBANKER in − 80 °C.

### Immunocytochemical analysis

Cells were fixed with 4% paraformaldehyde (PFA) in PBS for 10 min at 4 °C. After washing with PBS and treatment with 0.1% Triton X-100 (Sigma-Aldrich, #T8787-100 ML) for 10 min at 4 °C, the cells were incubated with Protein Block Serum-Free Ready-To-Use (Dako, #X 0909) for 30 min at room temperature, followed by reaction with primary antibody in blocking buffer for 24 h at 4 °C. After washing with PBS, the cells were incubated with fluorescently conjugated secondary antibody. Anti-rabbit or anti-mouse immunoglobulin G (IgG) bound to Alexa 488 or 546 (1:1000) was incubated in blocking buffer for 30 min at room temperature. The nuclei were stained with DAPI (Biotium, #40043). All images were captured using confocal microscopy (confocal microscope C2+) or fluorescence microscopy (BZ-X700, KEYENCE). Antibody information is provided in Table 2.

**Table 2:** List of antibodies for immunochemistry

Name	Class	Company	Dilution
Primary antibodies			
Vimentin (D21H3) XP #5741	Rabbit IgG	CST	1/100
Anti-pan Cytokeratin antibody [AE1/AE3 + 5D3] ab86734	Mouse IgG1	abcam	1/100
Anti-Estrogen Receptor alpha antibody [ESR1/3557] ab259427	Mouse IgG1	abcam	1/200
Anti-Progesterone Receptor antibody [SP2] ab16661	Rabbit IgG	abcam	1/100
Anti-CD10 antibody [EPR22865-73] ab255609	Rabbit IgG	abcam	1/50
Anti-CD13 antibody [EPR4058] ab108310	Rabbit IgG	abcam	1/100
Anti-E Cadherin antibody [HECD-1] ab1416	Mouse IgG1	abcam	1/50
Secondary antibodies			
DAPI #40043	None	Biotium	1/1000
Goat anti-rabbit IgG Secondary antibody, Alexa Fluor 488 A11008	None	Invitrogen	1/500
Goat anti-mouse IgG1 Secondary antibody, Alexa Fluor 546 A21123	None	Invitrogen	1/500

DAPI, 4′,6-Diamidino-2-Phenylindole, dihydrochloride

### Decidualization

For decidualization, endometrial stromal cells were plated in 6-well plates, then the cells were cultured for 8 days in DMEM supplemented with low-serum medium (2% FBS), 10 nM β-estradiol (E2758, Sigma-Aldrich, Saint Louis, MO, USA), 1 μM progesterone (E8783, Sigma-Aldrich, Saint Louis, MO, USA), and 0.5 mM 8-Br-cAMP (B5386, Sigma-Aldrich, Saint Louis, MO, USA). Detail protocol is shown in Supplemental Figure 1.

### Real-time quantitative polymerase chain reaction

RNA was extracted from cells using the RNeasy Mini kit (Qiagen, #74104). An aliquot of total RNA was reverse-transcribed using an oligo (dT) primer (Invitrogen, #18418-020). For the thermal cycle reactions, the cDNA template was amplified (Applied Biosystems Quantstudio 12 K Flex Real-Time PCR System) with gene-specific primer sets (Table 3) using the Platinum SYBR Green qPCR SuperMix-UDG with ROX (Invitrogen, #11733-046) under the following reaction conditions: 40 cycles of PCR (95 °C for 15 s and 60 °C for 1 min) after an initial denaturation (95 °C for 2 min). Fluorescence was monitored during every PCR cycle at the annealing step. mRNA levels were normalized using glyceraldehyde-3-phosphate dehydrogenase as a housekeeping gene.

**Table 3:** List of primers for quantitative reverse transcription-polymerase chain reaction

Gene	Primer sequence	
PRL	Forward	5′ TCATCTGGTCACGGAAGTACGT 3′
	Reverse	5′ GCCCTCTAGAAGCCGTTTGG 3′
IGFBP1	Forward	5′ ATGGCTCGAAGGCTCTCCAT 3′
	Reverse	5′ TCCTGTGCCTTGGCTAAACTC 3′
GAPDH	Forward	5′ TGTTGCCATCAATGACCCCTT 3′
	Reverse	5′ CTCCACGACGTACTCAGCG 3′

*PRL* prolactin, *IGFBP1* insulin-like growth factor binding protein 1, *GAPDH* glyceraldehyde 3-phosphate dehydrogenase

### Preparation of mouse embryonic fibroblasts

Mouse embryonic fibroblasts (MEF) were prepared for use as nutritional support cells (feeder cells). E12.5 ICR mouse fetuses (Japan CLEA) were excised and the fetus head, limbs, tail, and internal organs were all removed, minced with a blade, and seeded in culture dishes in a medium (DMEM containing 10% FBS, 1% Penstrep.) to allow cell growth. X-ray irradiation was applied (Hitachi, MBR-1520 R-3) to the cells in 1/100 amount of 1 M HEPES Buffer Solution (Invitrogen, 15630-106). Following irradiation with X-rays (dose, 30 Gy), the cells were frozen using a TC protector (DS Pharma Biomedical, TCP-001) and subsequently used as feeder cells for culturing endometrial epithelial cells.

### Preparation of human embryonic stem cell-derived feeder cells

The human embryonic stem cell line (SEES5) was maintained on irradiated MEF feeder layers [15]. To prepare human embryonic stem cell (hESC)-derived feeder cells, we processed SEES5 cells as previously reported [16]. Briefly, to generate embryonic bodies (EBs), SEES5 (5 × 10^3^/well) were dissociated into single cells with 0.5 mM EDTA (Life Technologies) after exposure to the rock inhibitor (Y-27632: A11105-01, Wako, Japan) and cultivated in 96-well plates (Thermo Fisher Scientific) in the EB medium (76% Knockout DMEM, 20% 35 kGy irradiated Xeno-free Knockout Serum Replacement (XF-KSR, Life Technologies, CA, USA), 2 mM GlutaMAX-I, 0.1 mM NEAA, Pen-Strep, and 50 μg/ml l-ascorbic acid 2-phosphate (Sigma-Aldrich, St. Louis, MO, USA)) for 4 days. The EBs were transferred to T25 flasks coated with NMP collagen PS (Nippon Meat Packers Inc.) and cultivated in the XF32 medium (85% Knockout DMEM, 15% 35 kGy-irradiated XF-KSR, 2 mM GlutaMAX-I, 0.1 mM NEAA, Pen-Strep, 50 μg/ml l-ascorbic acid 2-phosphate, 10 ng/ml heregulin-1β (recombinant human NRG-beta 1/HRG-beta 1 EGF domain; Wako, Japan), 200 ng/ml recombinant human IGF-1 (LONGR3-IGF-1; Sigma-Aldrich), and 20 ng/ml human bFGF (Kaken Pharmaceutical Co. Ltd.)) for 60 to 70 days. We propagated them in α-MEM medium supplemented with 10% FBS (Gibco or HyClone) and 1% Pen-Strep.

These cells were immortalized by inoculation with CSII-CMV-TERT, CSII-CMV-Tet-Off, CSII-TRE-Tight-cyclin D1, and CSII-TRE-Tight-CDK4R24C (mutant CDK4: an INK4a-resistant form of CDK4) lentiviruses according to our previous report [17]. We further infected the immortalized cells with the lentiviruses carrying a combination of the *R-WNT3A*, *R-SPO*, and *NOGGIN* genes. We designated the hESC-derived feeder cells (hESCFCs) and named as listed in Supplemental Table 1. We propagated these cells in a gelatin (Sigma, G1890)-coated culture dish in α-MEM medium supplemented with 10% FBS (Gibco) and 1% Pen-Strep to allow cell growth. For hESCFC-3 cultivation, we further added tetracycline into the medium. Using an X-ray irradiation apparatus (Hitachi, MBR-1520 R-3), 1/100 amount of 1 M HEPES Buffer Solution (Invitrogen, 15630-106) was added to the feeder cells except for hESCFC-3. Following irradiation with X-rays (dose, 30 Gy), the feeder cells were frozen using a TC protector (DS Pharma Biomedical, TCP-001). We did not use X-ray irradiation for hESCFC-3, and these cells were also frozen using a TC protector.

### Culture of endometrial epithelial cells

Endometrial epithelial cells were cultured on feeder cells. We cultured the endometrial epithelial cells for approximately 2 weeks and then passaged them when they reached confluency or ceased colony enlargement. We continued to observe growth of small colonies and terminated the culture when the cells did not show the signs of proliferation. In serial passages, the feeder cells were detached from culture dishes 1–2 min after exposure to TrypLE Express, but colonies of endometrial epithelial cells remained attached on the dish. MEFs and hESCFCs were cultured on gelatin-coated dishes in α-MEM supplemented with 10% FBS (Gibco) and 1% Pen-Strep as feeder cells. Endometrial stromal cells were cultured in DMEM (Gibco) supplemented with 10% FBS (Gibco) and 1% Pen-Strep. The endometrial epithelial cells were overlaid on the feeder cells in ESTEM-HE medium (GlycoTechnica, Japan). The media were replaced in 2 or 3 days.

### Assessment of cell proliferation

Endometrial epithelial cells were plated into 24 well plates (10 × 10^4^/well). In each well, corresponding feeder cells were plated in advance (5 × 10^4^/well). Cell clusters appeared 2 to 3 days after seeding, and the medium was replaced every 2 or 3 days. Phase-contrast photomicrographs were taken at each passage when the cells reached sub-confluence or stopped growing. Numbers of cells and colonies were counted in central field of pictures to compare feeder activities. Two investigators (R. Y. and Y.F) counted the total number of endometrial epithelial cells/well and the number of colonies formed, and calculated the area of colonies using imaging software Fiji [18]. These measurements were conducted entirely independent of all other variables. Each experiment was done in triplicate.

### Growth curves

Endometrial stromal cells were plated into 6-well plates (1 × 10^5^/well). The total number of cells/well, which was cultured in DMEM and ESTEM-HE medium, was counted 1, 3, 5,7, and 10 days after the plating.

### Three-dimensional cell culture

Three-dimensional cell culture was performed according to a previously described protocol [19]. Atelocollagen (Koken, #IPC-50) and endometrial stromal cells were mixed and poured into an untreated 60-mm Petri dish and allowed to gel at 37 °C for 1 h to prepare the stromal layer. Contraction of the collagen gel was facilitated by pulling the gel from the surface of the Petri dish. The medium (DMEM) was changed every 2 or 3 days until day 7. Frozen-thaw endometrial epithelial cells were plated at 1 × 10^6^cells inside a glass ring (10 mm diameter) on the surface of the contracted collagen gel at Day 7. Endometrial epithelial cells were grown in ESTEM-HE medium and the medium was replaced every 2 or 3 days. Three-dimensional endometrium was obtained on day 21.

### Statistical analysis

Changes in gene expression were compared by paired *t* tests. Unpaired Student’s *t* test was used to compare differences of continuous variables in two groups and one-way ANOVA was used in three groups. Prism 8.01 software (GraphPad Inc.) was used for the statistical analyses. *P* < 0.05 was considered to be statistically significant for all analyses.

## Results

### Endometrial epithelium and stroma

**Fig. 1:**
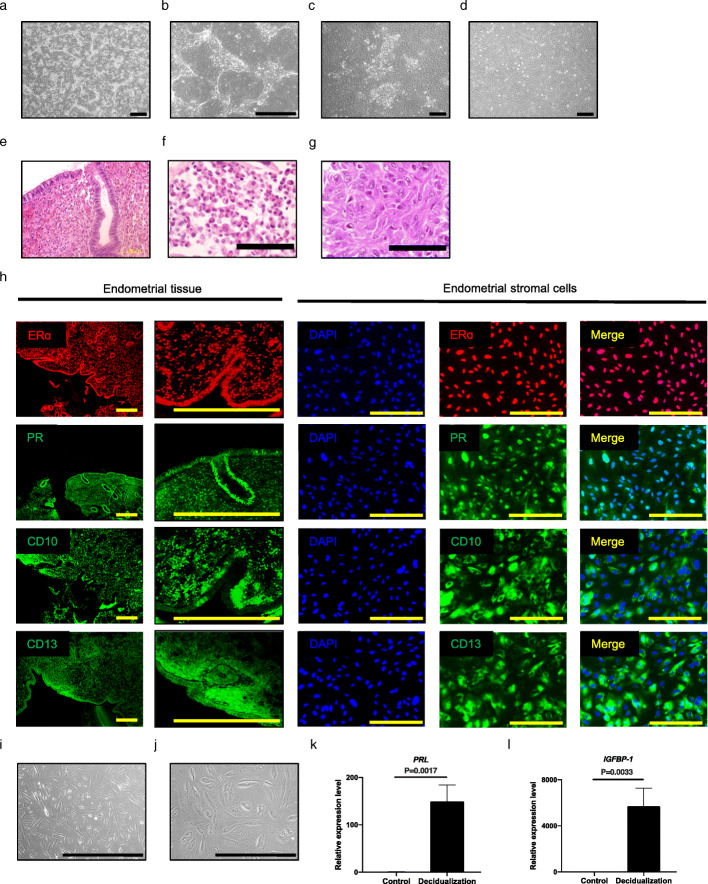
Preparation of endometrial epithelial cells and stromal cells from endometrial specimens. **a**, **b** Microscopic appearance of endometrial epithelial cells in primary culture. Low magnification (**a**) and high magnification (**b**). Black bar is 500 μm. **c**, **d** Microscopic appearance of endometrial stromal cells. Epithelial components were mixed in primary culture (**c**); however, these disappeared with serial passage (**d**). Black bar is 500 μm. **e**–**g** Hematoxylin and eosin staining for endometrial tissue (**e**), cultured endometrial epithelial cells (**f**), and stromal cells (**g**). Black bar is 100 μm. **h** Immunohistochemical staining for endometrial stromal cells in serial culture. Endometrial tissue was used as a control. Endometrial stromal cells were positive for ERɑ, PR, CD10, and CD13 like endometrial tissue. Nuclei were stained with DAPI. Yellow bar is 500 μm. Epithelial component of endometrial tissue was positive for ERɑ and PR, negative for CD10, and weakly positive for CD13. **i**–**l** Decidualization of endometrial stromal cells. Microscopic appearance of endometrial stromal cells cultured in the control medium (**i**) and supplemented with estrogen, progesterone, and cAMP (**j**) (see Experimental procedure). Black bar is 500 μm. The *PRL* (**k**) and *IGFBP-1* (**l**) genes were significantly upregulated after decidualization (*P* = 0.0017 and 0.0033, respectively). Expression of the genes in the controls is designated as 1.0. Error bar indicates SEM. Each experiment was done in triplicate. Abbreviation: ER, estrogen receptor; PR, progesterone receptor; DAPI, 4′,6-diamidino-2-phenylindole; cAMP, cyclic adenosine monophosphate; PRL, prolactin; IGFBP-1, insulin-like growth factor binding protein-1; SEM, standard error of the mean

We collected endometrial samples from healthy women undergoing benign gynecological surgery and prepared primary human endometrial epithelial cells and stromal cells from the endometrial samples by enzymatic digestion for in vitro experiments (Table 1, Fig. 1a–d). During culture of stromal cells, epithelial cells were observed at early passages; however, they disappeared after serial passages (Fig. 1c, d). We failed to culture the endometrial cells from the endometrial samples derived from older patients and patients in the secretory phase of their cycles because the cells ceased proliferating. Endometrial cells may have differential growth properties that depend on donor age and menstrual phase.

We then examined the thawed endometrial cells to investigate whether endometrial characteristics were preserved (Fig. 1e–g). Endometrial cells in culture exhibited a single columnar epithelium structure and spindle-shaped fibroblast-like morphology that were similar to normal endometrial tissue. For endometrial stromal cells, we further explored protein expression and hormone responsiveness (Fig. 1h–l). Endometrial stromal cells presented the same protein expression pattern as endometrial tissue. We successfully induced decidualization in endometrial cells which exhibited aggregation of large cells with abundant cytoplasm, marked nucleolar enlargement and prominent nuclei (Fig. 1i, j; Supplemental Figure 1). Additionally, the decidualization markers *PRL* and *IGFBP-1* were significantly upregulated in the decidualized cells (*P* = 0.0017 and 0.0033, respectively) (Fig. 1k, l), suggesting that hormone responsiveness is preserved in endometrial stromal cells even after the freeze-thaw procedure and subsequent passages.

### Successful cultivation of endometrial epithelial cells with feeder cells

**Fig. 2:**
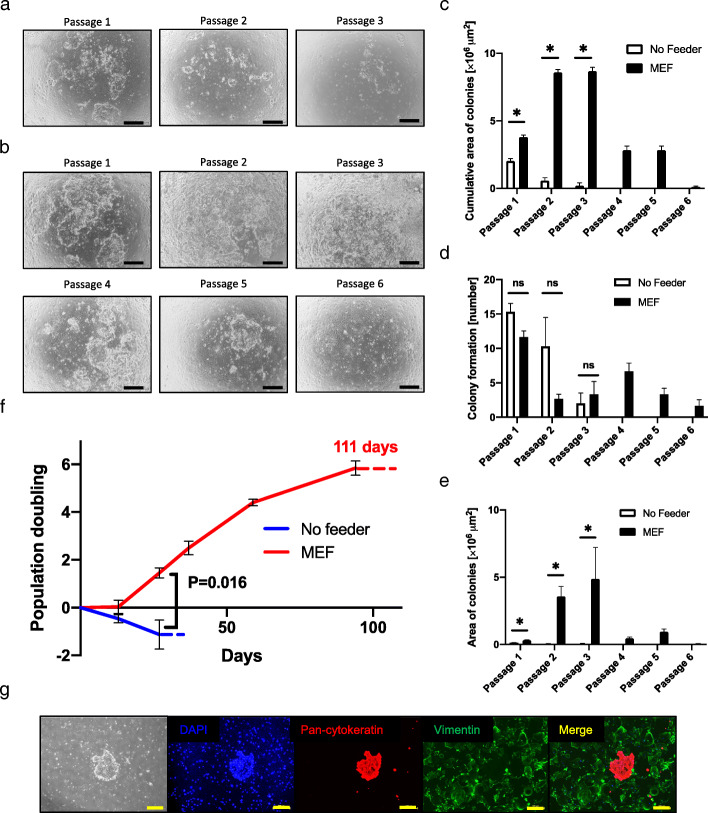
Culture of endometrial epithelial cells with mouse embryonic fibroblasts. **a**, **b** Microscopic appearance of endometrial epithelial cells without feeder cells (**a**) and with MEF (**b**) in serial passage. Black bar is 500 μm. **c**–**e** Cumulative area of colonies (**c**), colony formation (number) (**d**), and area of colonies (**e**) of endometrial epithelial cells in serial passages. Error bar indicates SEM. An asterisk means *P* < 0.05. ns means “not significant”. **f** Population doubling levels of endometrial epithelial cells when culture with MEF (red) and without feeder cells (blue). We could propagate endometrial epithelial cells with MEF for 111 days. Error bar indicates SEM. Dotted line indicated the observation period until the culture was terminated. **g** Immunohistochemical staining for endometrial epithelial cells and MEF at passage 4. Endometrial epithelial cells kept positive for pan-cytokeratin in serial passage. MEF expressed vimentin. Endometrial epithelial cells did not express vimentin. Nuclei were stained with DAPI. Yellow bar is 500 μm. Each experiment was done in triplicate. Abbreviation: MEF, mouse embryonic fibroblasts; SEM, standard error of the mean

We then examined various cells as feeders for endometrial epithelium (Fig. 2a–f). We serially passaged endometrial epithelial cells on mouse embryonic fibroblasts (MEF) up to 6 passages for approximately 4 months (Fig. 2b). Since the adhesive ability of MEFs was low, MEFs were easily separated from the endometrial epithelial cells. The endometrial epithelial cells were efficiently propagated on MEFs, as shown by the cumulative area of colonies (*P* < 0.05), area of colonies (*P* < 0.05), and population doubling (*P* = 0.016) (Fig. 2a–f). Endometrial epithelial cells on MEF showed a cobblestone appearance and expressed pan-cytokeratin, an epithelial cell-specific marker, at passage 4 (Fig. 2g, Supplemental Figure 2A). Interestingly, endometrial epithelial cells on MEF lost expression of vimentin, which is expressed in both endometrial epithelial cells and stromal cells.

### Patient-derived endometrial stromal cell is one of the best human feeder cells

**Fig. 3:**
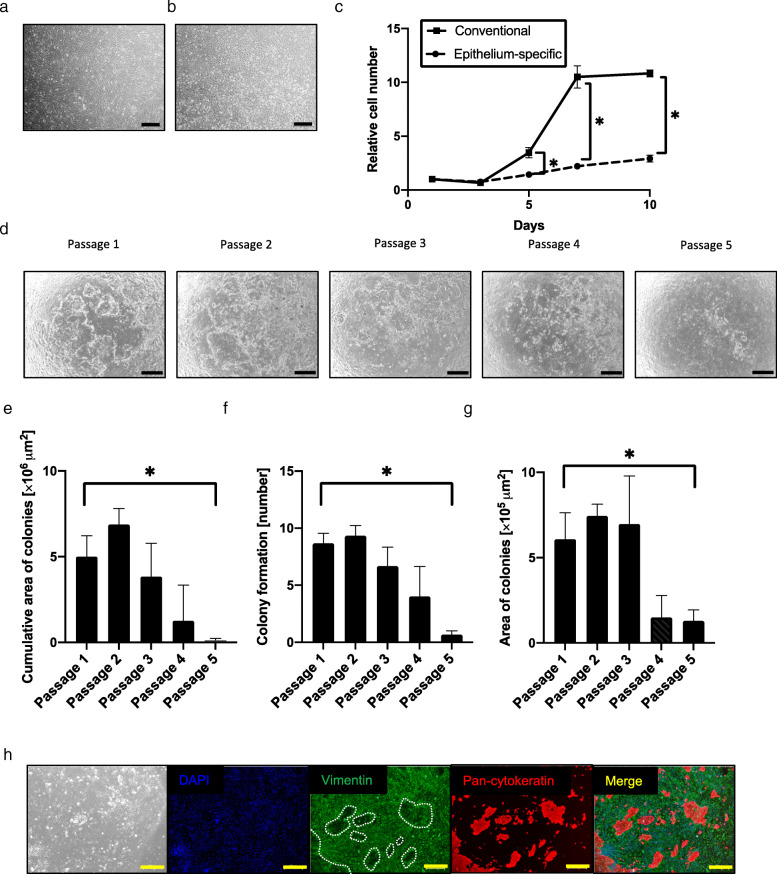
Culture of endometrial epithelial cells with endometrial stromal cells. **a**, **b** Microscopic appearance of endometrial stromal cells cultured in conventional medium (DMEM) (**a**) and epithelium-specific medium (ESTEM-HE medium) (**b**). Black bar is 500 μm. **c** Growth curves of endometrial stromal cells cultured in conventional and epithelium-specific medium. Error bar indicates SEM. An asterisk means *P* < 0.05. **d** Microscopic appearance of endometrial epithelial cells with endometrial stromal cells in serial passage. Black bar is 500 μm. **e**–**g** Cumulative area of colonies (**e**), colony formation (number) (**f**), and area of colonies (**g**) of endometrial epithelial cells in serial passage with endometrial stromal cells. Error bar indicates SEM. An asterisk means *P* < 0.05. **h** Immunocytochemical staining for endometrial epithelial cells and endometrial stromal cells at passage 4. Endometrial epithelial cells (surrounded with white dotted lines) continued to express pan-cytokeratin, but not vimentin, at passage 4. Endometrial stromal cells were positive for vimentin. Nuclei were stained with DAPI. Yellow bar is 500 μm. Each experiment was done in triplicate. Abbreviation: DMEM, Dulbecco’s modified Eagle’s medium; SEM, standard error of the mean

Anticipating clinical applications, we examined human cells as feeder cells for endometrial epithelial cells. We assumed that endometrial stromal cells simultaneously obtained from the same patient would be good feeders for endometrial epithelial cells. We hypothesized that endometrial stromal cells could serve as feeder cells if their proliferation activities were suppressed. We therefore investigated differences in cell proliferation of endometrial stromal cells in two types of media. Endometrial stromal cells in proliferative and non-proliferative states were spindle-shaped or flat polygonal-shaped, respectively (Fig. 3a, b). The proliferation rate of endometrial stromal cells in the conventional medium (DMEM supplemented with 10% FBS) was significantly higher than that in the epithelium-specific medium (*P* < 0.05) (Fig. 3c). Based on these findings, we decided to utilize the endometrial stromal cells as feeder cells for subsequent experiments. The endometrial epithelial cells were propagated on endometrial stromal cells up to passage 5 for 81 days (Fig. 3d–g). Estrogen receptor α and progesterone receptor were preserved in the endometrial epithelial cells (Supplemental Figure 3A and B). Endometrial epithelial cells after serial passages exhibited a cobblestone appearance and expressed pan-cytokeratin, but they failed to express vimentin at early passage (passage 2) and late passage (passage 4) (Fig. 3h, Supplemental Figure 3C).

**Fig. 4:**
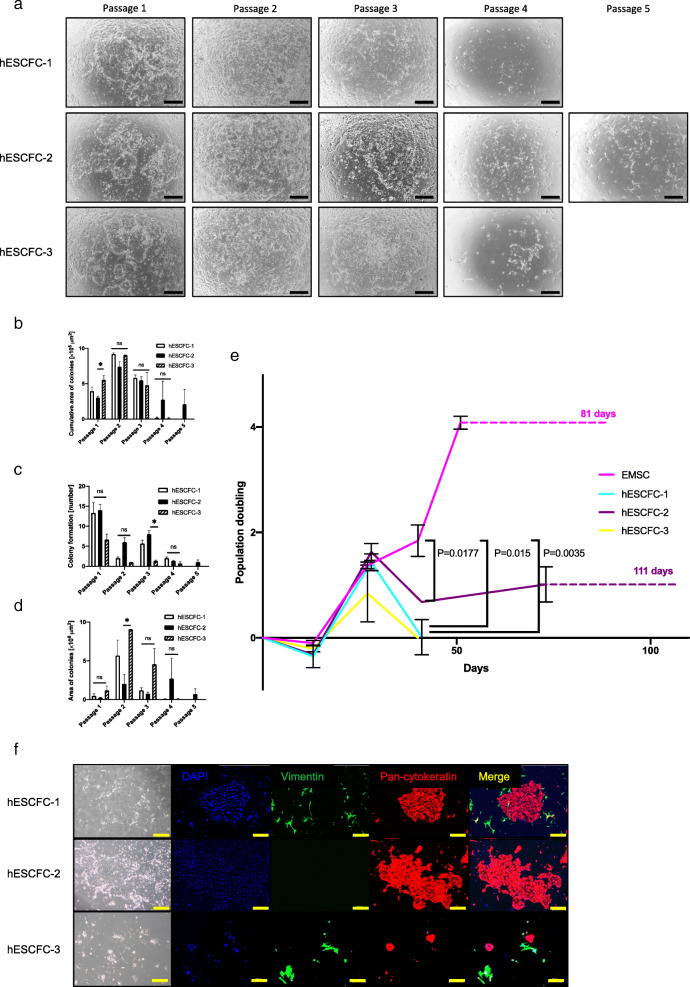
Culture of endometrial epithelial cells with human embryonic stem cell-derived mesenchymal cells. **a** Microscopic appearance of endometrial epithelial cells with hESCFCs (hESCFC-1, 2, 3) in serial passage. Black bar is 500 μm. **b**–**d** Cumulative area of colonies (**b**), colony formation (number) (**c**), and area of colonies (**d**) of endometrial epithelial cells in serial passages with hESCFCs. Error bar indicates SEM. An asterisk means *P* < 0.05. ns means “not significant”. **e** Population doubling levels of endometrial epithelial cells when cultured with endometrial stromal cells and hESCFCs. Endometrial stromal cells showed the best feeder activities among these feeder cells (*P* = 0.015 when comparing with hESCFC-1, *P* = 0.0177 when compared with hESCFC-2, and *P* = 0.0035 when comparing with hESCFC-3). Endometrial epithelial cells continued to proliferate on endometrial stromal cells for 81 days. Error bar indicates SEM. Dotted line indicated the observation period until the culture was terminated. **f** Immunocytochemical staining for endometrial epithelial cells and hESCFCs at passage 4. Endometrial epithelial cells kept positive for pan-cytokeratin with serial passage. The endometrial epithelial cells did not express vimentin. hESCFCs expressed vimentin. Nuclei were stained with DAPI. Yellow bar is 500 μm. Each experiment was done in triplicate. Abbreviation: EMSC, endometrial stromal cells; hESCFCs, human embryonic stem cell-derived feeder cells; SEM, standard error of the mean

We then examined human-derived cells (hESCFCs) as feeder cells for endometrial epithelial cells. We simultaneously compared feeder activities of endometrial stromal cells and hESCFCs by examination of the proliferation rate of endometrial epithelial cells under the same experimental conditions, including cell density and culture media. The endometrial epithelial cells were successfully cultured on hESCFCs up to passage 5 for 4 months (Fig. 4a–e). Furthermore, we examined the endometrial epithelial cells for morphology and protein expression (Fig. 4f). The endometrial epithelial cells expressed pan-cytokeratin and lost expression of vimentin when cultured with hESCFCs. The proliferation rate of endometrial epithelial cells on the endometrial stromal cells was significantly higher than that on hESCFCs (*P* < 0.05). Endometrial stromal cells can therefore be used as feeder cells to support proliferation of endometrial epithelial cells, as they were among the best human-derived cells tested.

### Three-dimensional culture of thawed endometrial cells

**Fig. 5:**
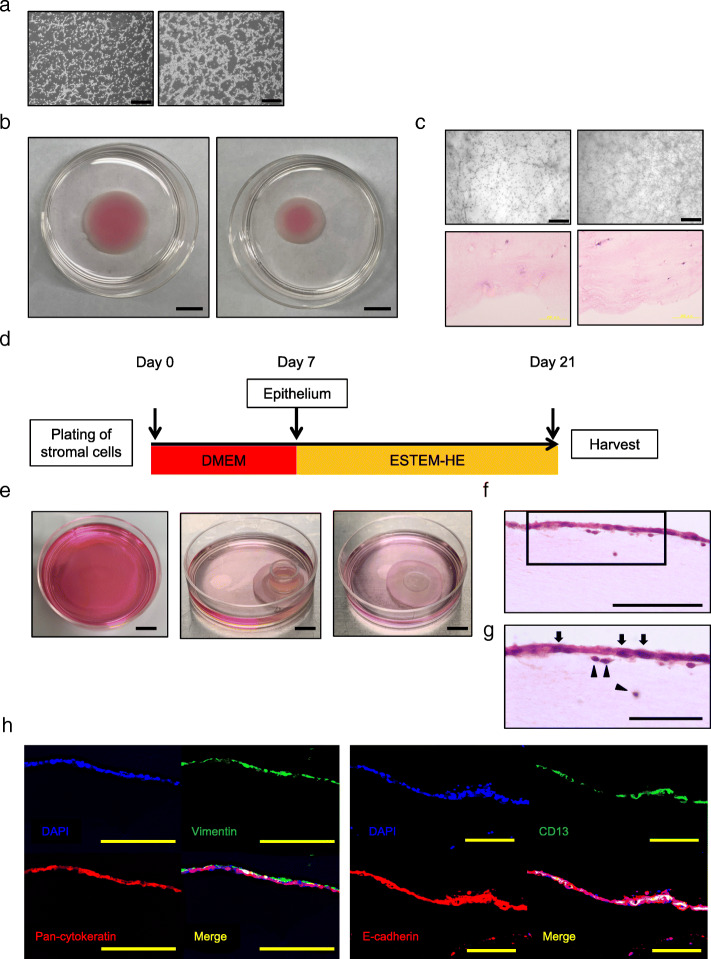
Endometrial three-dimensional cell culture model. **a** Microscopic appearance of endometrial stromal cells embedded in atelocollagen on Day 1. Cell numbers were 1 × 10^6^cells (left) and 2 × 10^6^cells (right), respectively. Black bar is 500 μm. **b** Gross appearance of endometrial stromal cells embedded in atelocollagen on day 7. Cell numbers were 1 × 10^6^cells (left) and 2 × 10^6^cells (right), respectively. Black bar is 1 cm. **c** Microscopic appearance (bright field and HE staining) of endometrial stromal cells embedded in atelocollagen on day 7. Cell numbers were 1 × 10^6^cells (left) and 2 × 10^6^cells (right), respectively. Black bar is 1 cm. **d** Protocol for three-dimensional cell culture. **e** Gross appearance of endometrial three-dimensional cell culture. Endometrial stromal cells were embedded in atelocollagen (left), then endometrial epithelial cells were plated on formed stromal layers using a glass ring on day 7 (middle). Endometrial three-dimensional model was developed during further 14 days of culture (right). **f** HE staining for three-dimensional cultured endometrial cells. Black bar is 100 μm. **g** Magnification of box area in figure **f**. Endometrial epithelial cells (arrows) and stromal cells (arrowheads) in three-dimensional cell culture. Black bar is 50 μm. **h** Immunohistochemistry of endometrial cells in three-dimensional culture. The endometrial epithelial cells were positive for pan-cytokeratin, vimentin, E-cadherin, and CD13. This is consistent with expression of these markers in intact endometrial tissue. Nuclei were stained with DAPI. Yellow bar is 200 μm

Our successful cultivation of endometrial epithelial cells for use in co-cultures with endometrial stromal cells motivated us to investigate whether three-dimensional culture can be achieved using thawed endometrial cells. We investigated whether variation in the numbers of endometrial stromal cells in the atelocollagen gel affects three-dimensional-culture (Fig. 5a–c). Construction of artificial endometrium network depended on the number of endometrial stromal cells. Endometrial stroma was evenly embedded in the atelocollagen gel. Endometrial stromal cells (1 × 10^6^cells) embedded in atelocollagen formed stromal layer, and gradually shrunk during 7 days of culture (Fig. 5d). We then plated endometrial epithelial cells on formed stromal layers and maintained the three-dimensional-culture for 14 days (Fig. 5e–g). Epithelial cells in three-dimensional-culture were positive for both epithelial markers (cytokeratins and E-cadherin) and mesenchymal markers (vimentin and CD13), like intact human endometrium (Fig. 5h, Supplemental Figure 2B). These data suggest that endometrial epithelium and stroma, after the freeze-thaw procedure and sequential culture, are able to establish an endometrial three-dimensional model. The success of this study may lead to the development of an in vitro implantation model.

## Discussion

It is difficult to maintain endometrial epithelial cells in vitro and co-culture them with endometrial stromal cells. Similar three-dimensional-structures have been established in the cornea, intestine, and liver [20–22]. Likewise, we hypothesized that an endometrial three-dimensional model can be established. In this study, we demonstrated that endometrial stroma is one of the best feeder cell types for propagation of endometrial epithelium. We also established an endometrial three-dimensional model with frozen-thaw endometrial epithelial cells and endometrial stromal cells.

### Endometrial stromal cells as feeder cells

Feeder cells have the capacity to support in vitro survival and growth of orthologous epithelial or parenchymal cells through a variety of soluble or membrane-bound growth factors and receptors [23–25]. Functional epithelial and parenchymal cell types are dependent on physical contact with feeder cells for survival and expansion. On the other hand, feeder-dependent cells can also be grown under feeder-free conditions when coated with extracellular matrix proteins such as laminin, vitronectin, or a mixture of the extracellular matrix components [26–29]. Feeder cells usually consist of adherent growth-arrested, but viable cells. It may be necessary to maintain feeder cells in a nonmultiplying state by irradiation or exposure to anticancer drugs to prevent overgrowth [23]. This is observed in other types of feeder cells such as MEFs and immortalized feeder cells [30–35]. Irradiation of the feeder cells, i.e., MEFs, hESCFC-1 cells, hESCFC-2 cells, and hESCFC-3 cells with an inducible Tet-ON system, indeed enabled us to establish the endometrial model, implying that endometrial epithelial cells are supported by non-dividing endometrial stromal cells, but not endometrial stromal cells retaining proliferation activities. This finding motivated us to use endometrial stromal cells as feeder cells. Indeed, this study showed that endometrial stromal-derived cells are able to support long-term survival and growth of endometrial epithelial cells. We also investigated feeder activities of hESCFCs established in our laboratory. hESCFCs were easy to maintain. Furthermore, other types of human cells such as epidermal cells, intestinal epithelial cells, and hepatocytes can be propagated on hESCFCs in our laboratory (not published). These results motivated us to investigate whether hESCFCs can accelerate proliferation of endometrial epithelial cells. As for the differences among the feeder cells, various combinations of the genes have been transduced to investigate which genes contribute to good feeder activities. hESCFCs contribute the small success to propagate the endometrial epithelial cells. However, the difference among hESCFCs was subtle; Wnt3a and Rspo1 are sufficient to confer efficient feeder activity to hESCFCs. Further studies will be needed to clarify which genes are necessary to support proliferation of not only endometrial epithelial cells, but also other types of human epithelial or parenchymal cells.

The underlying mechanisms for the successful expansion of endometrial epithelium on homologous stroma developed in this study were not revealed; however, previous reports suggest that the FGF-MAPK, WNT-R-spondin-3, BMP-Noggin, and TGFβ signaling pathways are involved [12, 13, 36]. The success of endometrial epithelial cell proliferation via cell interaction is attributed to four mechanisms: First, endometrial stromal cells secrete signal molecules that act as local mediators, affect cells in the immediate environment, and exert a paracrine effect on endometrial epithelial cells [37]. Second, cell membrane-bound molecules of endometrial stromal cells influence endometrial epithelial cells [38]. Third, endometrial stromal cells contribute to the stabilization of the culture environment by producing extracellular matrices [39]. Fourth, the heterocellular contribution of endometrial stromal cells to endometrial epithelial cells may be through gap junctions [40].

### Potential clinical application

The culture method developed in this study has four strengths that suggest the cells will be useful for clinical applications. First, we employed a simplified culture method which acquires more viable cells without usage of magnetic cell sorting and/or fluorescence-activated cell sorting. Second, patient-derived endometrial stromal cells can be substituted for Matrigel. Third, we show proof-of-concept regarding the use of frozen cells; we successfully utilized thawed endometrial cells that were cultured for more than 3 months. Indeed, cryopreservation of freshly biopsied tissue was challenged [41]. These findings are critical for clinical application from the viewpoint that reproductive endocrinologists prepare patient-derived endometrial cells for ongoing fertility treatment in a clinical setting. Fourth, endometrial stromal cells served the best condition for endometrial epithelial cells in vitro. Epithelial cells in vivo require close interaction with surrounding mesenchymal cells [23]. To solve the problem of thin endometrium with regenerative medicine, we used endometrial somatic cells as a source of epithelial cells and feeder cells. Alternatively, endometrium-derived pluripotent stem cells and progenitors may also be an attractive source [42]. Although refinement of the protocol and proof-of-concept by in vivo experimentation are needed before applying these cells in clinical practice, findings from our study can lead to development of novel therapeutic strategies in fertility medicine.

## Conclusions

Our study demonstrates not only the promise for in vitro endometrial regeneration, but also advances our understanding of reproductive biology. Novel in vitro approaches, including modifying embryo culture so-called “in vitro implantation”, may be possible therapeutic approaches to increase success rates of fertility treatment and diminish unnecessary miscarriage.

## Supplementary Information

**Additional file 1: Supplemental Figure 1.** Protocol of decidualization. Control medium was DMEM with low-serum medium (2% FBS) and Penstrep. For decidualization, β-estradiol, progesterone and 8-Br-cAMP were added in control medium as supplement. Medium replacement (MR) was performed every other day.

**Additional file 2: Supplemental Figure 2.** Immunohistochemistry for endometrial tissues. (A) The epithelial component of endometrial tissue is positive for pan-cytokeratin and vimentin. Nuclei were stained with DAPI. Yellow bar is 200 μm. (B) The epithelial component of endometrial tissue is positive for E-cadherin and vimentin. Nuclei were stained with DAPI. Yellow bar is 200 μm.

**Additional file 3: Supplemental Figure 3.** Immunocytochemical staining for endometrial epithelial cells cultured on endometrial stromal cells at passage 2. A, B: Endometrial epithelial cells (surrounded with white dotted lines) remained positive for estrogen receptor α (A: ERα) and progesterone receptor (B: PR). C: Endometrial epithelial cells (surrounded with white dotted lines) were positive for pan-cytokeratin. Endometrial stromal cells expressed vimentin, but endometrial epithelial cells did not. Yellow bar is 100 μm.

**Additional file 4: Supplemental Table 1.** List of vectors and genes.

## Data Availability

The datasets used and/or analyzed during the current study are available from the corresponding author on reasonable request.
