# Supplementary figures and images for: Endometrial regeneration with endometrial epithelium: homologous orchestration with endometrial stroma as a feeder
Source: Stem Cell Res Ther. 2021 Feb 12;12:130. doi: 10.1186/s13287-021-02188-x (PMC7881492; doi:10.1186/s13287-021-02188-x)

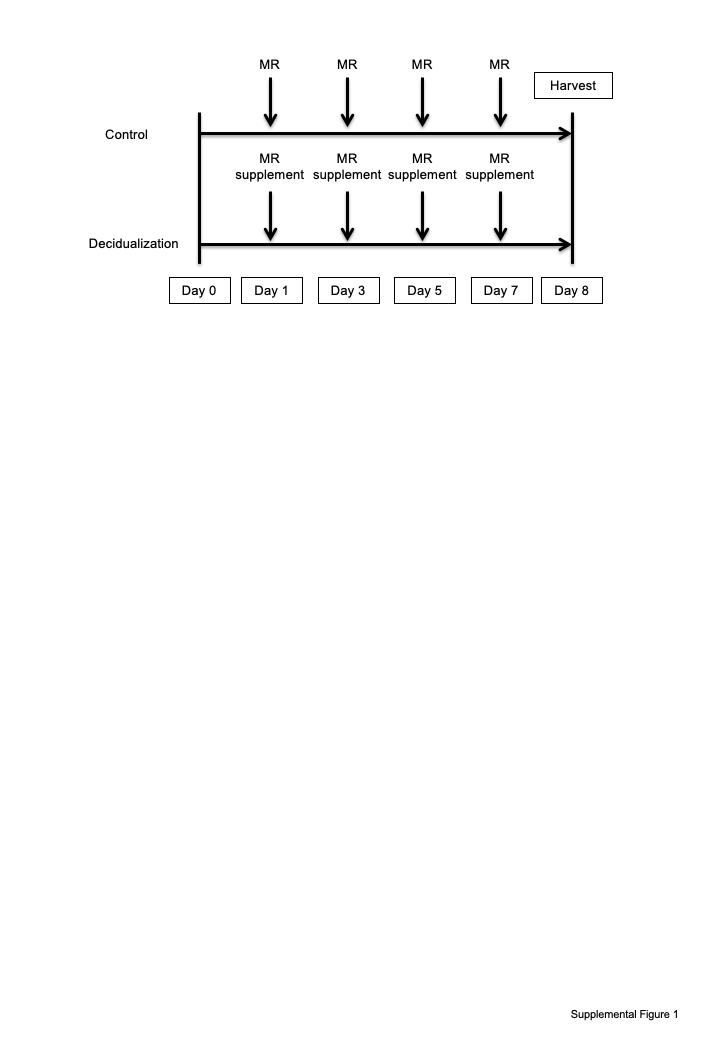

Supplement: Supplementary file 1 — Additional file 1: Supplemental Figure 1. Protocol of decidualization. Control medium was DMEM with low-serum medium (2% FBS) and Penstrep. For decidualization, β-estradiol, progesterone and 8-Br-cAMP were added in control medium as supplement. Medium replacement (MR) was performed every other day. [file 13287_2021_2188_MOESM1_ESM.tiff]

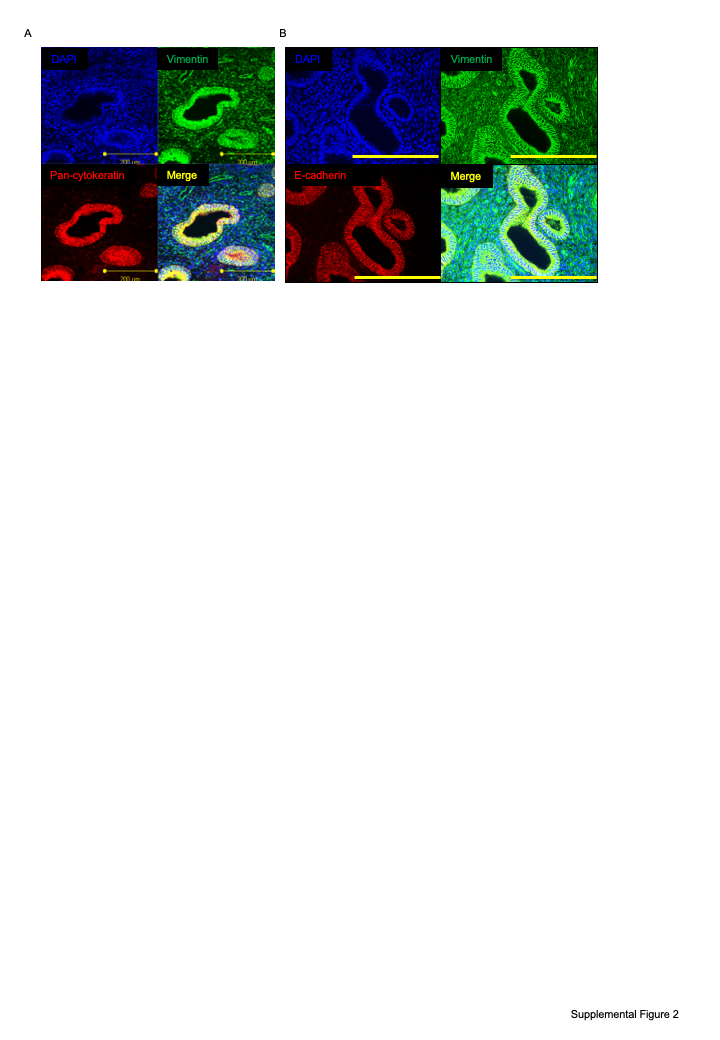

Supplement: Supplementary file 2 — Additional file 2: Supplemental Figure 2. Immunohistochemistry for endometrial tissues. (A) The epithelial component of endometrial tissue is positive for pan-cytokeratin and vimentin. Nuclei were stained with DAPI. Yellow bar is 200 μm. (B) The epithelial component of endometrial tissue is positive for E-cadherin and vimentin. Nuclei were stained with DAPI. Yellow bar is 200 μm. [file 13287_2021_2188_MOESM2_ESM.tiff]

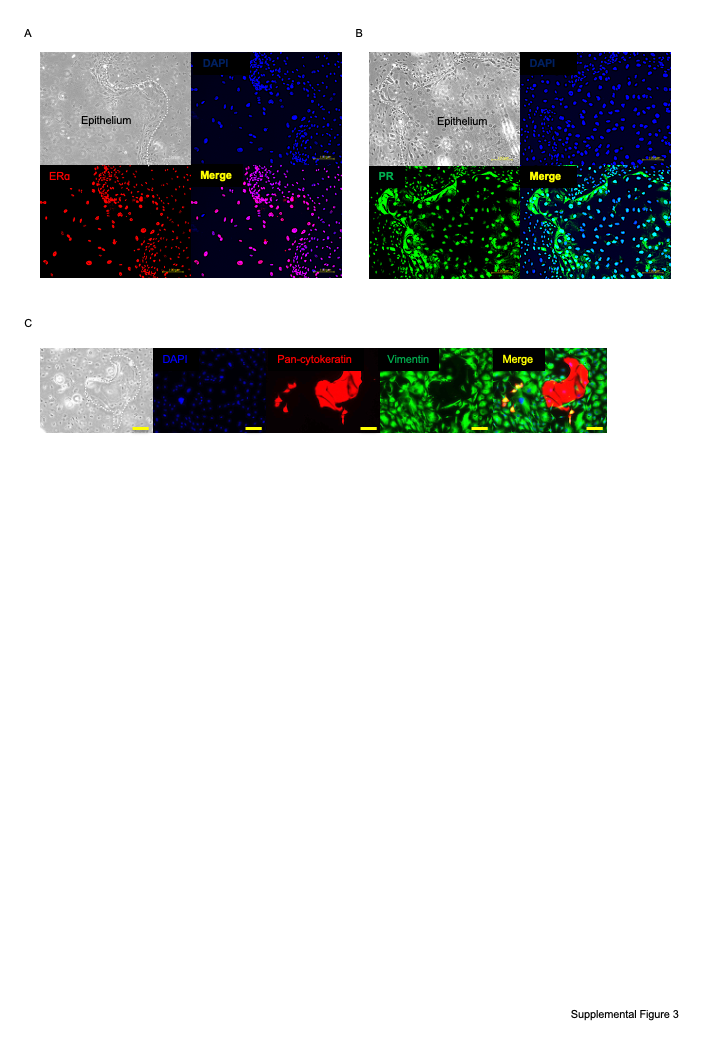

Supplement: Supplementary file 3 — Additional file 3: Supplemental Figure 3. Immunocytochemical staining for endometrial epithelial cells cultured on endometrial stromal cells at passage 2. A, B: Endometrial epithelial cells (surrounded with white dotted lines) remained positive for estrogen receptor α (A: ERα) and progesterone receptor (B: PR). C: Endometrial epithelial cells (surrounded with white dotted lines) were positive for pan-cytokeratin. Endometrial stromal cells expressed vimentin, but endometrial epithelial cells did not. Yellow bar is 100 μm. [file 13287_2021_2188_MOESM3_ESM.tiff]
