# Supplementary material for: Endometrial regeneration with endometrial epithelium: homologous orchestration with endometrial stroma as a feeder
Source: Stem Cell Res Ther. 2021 Feb 12;12:130. doi: 10.1186/s13287-021-02188-x (PMC7881492; doi:10.1186/s13287-021-02188-x)
Supplement: Supplementary file 4 — Additional file 4: Supplemental Table 1. List of vectors and genes. [file 13287_2021_2188_MOESM4_ESM.docx]

**Supplemental Table 1. List of transduced genes and vectors**

| Cell name | Original label | Transduced genes |
| --- | --- | --- |
| hESCFC-1 | iHep1 5-2（S-5-74） | WNT3A, R-SPO1 |
| hESCFC-2 | iHep1 5-2（Ⅱ-2-7） | WNT3A, R-SPO1, NOG |
| hESCFC-3 | iHep1 5-2（3-5） | WNT3A, R-SPO1, NOG |

*hESCFC, human embryonic stem cell-derived feeder cells

hESCFC-1: CSII-CMV-WNT3A, PQCXIP-AFM, PQCXIP-R-SPO1.

hESCFC-2: CSII-CMV-WNT3A, PQCXIP-AFM, PQCXIP-R-SPO1, PQCXIP-NOG.

hESCFC-3: CSII-CMV-WNT3A, PQCXIP-AFM, PQCXIP-R-SPO1, PQCXIP-NOG, PB-TACOff-ERN-SpCas9-GR+p11018:pCAG-hyperPBase(I30V/S103P/G165S/M282V/N538K/S509G/N570S)
